# PHYSIOLOGICAL DETERMINANTS OF ENDURANCE CAPACITY IN INDIVIDUALS WITH CEREBRAL PALSY: A NARRATIVE REVIEW

**DOI:** 10.2340/jrm.v58.46260

**Published:** 2026-10-06

**Authors:** Linnéa CORELL, Nina MOSSER, Sebastian EDMAN, Oscar HORWATH, Sudarshan DAYANIDHI, Emma HJALMARSSON, Jessica NORRBOM, Annika KRUSE, Ferdinand VON WALDEN

**Affiliations:** 1 Department of Women’s and Children’s Health, Division of Paediatric Neurology, Karolinska Institutet, Stockholm, Sweden; 2 Department of Human Movement Sciences, Sport and Health, University of Graz, Graz, Austria; 3 Department of Physiology, Nutrition and Biomechanics, The Swedish School of Sport and Health Sciences, Stockholm, Sweden; 4 Department of Physiology and Pharmacology, Karolinska Institutet, Stockholm, Sweden; 5 Shirley Ryan AbilityLab, Chicago, IL, USA; 6 Feinberg School of Medicine, Northwestern University, Chicago, IL, USA; 7 Theme Women’s Health and Allied Health Professionals, Medical Unit Allied Health Professionals, Karolinska University Hospital, Stockholm, Sweden

**Keywords:** cardiorespiratory fitness, cerebral palsy, exercise tolerance, physical activity, muscle, skeletal

## Abstract

**Objective:**

To synthesize evidence on the physiological determinants of endurance capacity in individuals with cerebral palsy.

**Design:**

Narrative review.

**Subjects/Patients:**

Children and adults with cerebral palsy across the spectrum of motor function (Gross Motor Function Classification System I–V).

**Methods:**

Relevant literature on cardiovascular, pulmonary, haematological, and musculoskeletal determinants of endurance capacity in individuals with cerebral palsy was identified and synthesized.

**Results:**

Reduced cardiorespiratory fitness is common, with autonomic dysregulation potentially constraining heart rate responses during exercise, while lower oxygen pulse suggests limitations in stroke volume and peripheral oxygen extraction. Restrictive ventilatory patterns, including reduced functional lung volumes, may further impair exercise capacity. High energetic demands during basic movements exacerbate fatigue and reduce engagement in physical activity. Skeletal muscle also exhibits structural and metabolic alterations, including reduced muscle volume, capillary density, and mitochondrial function, impairing oxygen utilization and energy production.

**Conclusion:**

Despite methodological heterogeneity, evidence consistently indicates that integrated central and peripheral factors constrain endurance capacity, with impairments more pronounced in individuals with greater functional limitations. Adapted training modalities, such as Frame Running, show promise in improving these outcomes. Understanding these physiological determinants is essential for developing individualized interventions to improve function, participation, health, and quality of life in individuals with cerebral palsy.

## LAY ABSTRACT

Cerebral palsy is the most common early-onset motor disability and affects movement and posture throughout life. Children and adults with cerebral palsy often have lower endurance than their typically developing peers, meaning that physical activities are more tiring and harder to sustain. This can limit daily activities and increase the risk of other health problems, such as cardiovascular and metabolic conditions. Here, we summarize current knowledge on why endurance is reduced in individuals with cerebral palsy. We describe how differences in heart, lungs, and muscles can limit exercise capacity, and how everyday movements often require more energy than usual, leading to early fatigue. Importantly, tailored exercise programmes and use of adaptive equipment, such as running frames, during exercise can help overcome many of these challenges. Understanding the factors affecting endurance can guide clinicians and therapists in developing personalized interventions that improve health, independence, and quality of life for individuals with cerebral palsy.

Cerebral palsy (CP) is a lifelong neurodevelopmental condition affecting approximately 1.6–3.4 in 1,000 live births globally (1). It is caused by malformation and/or an insult to the foetal or infant brain, resulting in impaired development of movement and posture (2, 3). Depending on the location and extent of the brain injury, functional limitations range from mild to severe and are typically classified using the Gross Motor Function Classification System (GMFCS; I–V) (4–6). While individuals classified at GMFCS I and II are generally independent ambulators, those at higher levels may require walking aids or rely on wheelchair mobility (5, 6).

Although the severity and manifestations vary considerably, CP is commonly associated with reduced physical activity and increased sedentary behaviour (7–9), contributing to an elevated cardiometabolic risk profile including elevated rates of hypertension, hypercholesterolaemia, visceral adiposity, and obesity in adulthood (10–14). Correspondingly, individuals with CP exhibit a two- to threefold higher mortality rate from coronary heart disease compared with the general population (15). Notably, cardiometabolic risk already appears elevated early in life and is associated with motor impairment severity and low physical activity levels in children and adolescents with CP (16–19).

Central to these health challenges is reduced endurance capacity – the ability to sustain physical activity over time – which depends on the efficiency of multiple organ systems (20). This capacity is fundamental to daily activities and closely linked to independence, participation, and quality of life (21–23). Cardiorespiratory fitness is a key determinant of endurance capacity, commonly assessed by measuring peak oxygen uptake (VO_2peak_) (24). VO_2peak_ is determined by both central and peripheral factors: cardiac output, pulmonary gas exchange efficiency, haemoglobin concentration and plasma volume for oxygen transport, as well as skeletal muscle oxidative capacity for oxygen extraction and utilization (20). Individuals with CP consistently demonstrate lower cardiorespiratory fitness compared with typically developing peers (25–30), a finding of considerable clinical relevance given strong associations between reduced cardiorespiratory fitness and adverse health outcomes (10–12).

Cardiorespiratory fitness tends to track from childhood into adulthood (31), underscoring the importance of early identification and intervention to optimize long-term health trajectories. Breaking the vicious circle of low physical activity and poor fitness is therefore essential. However, the degree and nature of impairments vary widely across individuals with CP, highlighting the need for individualized approaches to intervention and deeper understanding of the physiological factors that limit endurance capacity in this population.

Therefore, the aim of this review is to provide a comprehensive overview of the physiological determinants of endurance capacity in individuals with CP. We synthesize current evidence on central and peripheral physiological factors influencing endurance capacity, describe relevant testing modalities across functional levels, and identify knowledge gaps to guide future research.

## PHYSICAL ACTIVITY AND SEDENTARY BEHAVIOUR IN INDIVIDUALS WITH CEREBRAL PALSY

Individuals with CP consistently demonstrate reduced physical activity levels across the lifespan (32), with those with more pronounced impairments tending to be the least active (33–35). In addition, individuals with CP exhibit high levels of sedentary behaviour, defined as any waking activity with an energy expenditure ≤1.5 metabolic equivalents while sitting, reclining, or lying (36). Evidence indicates that children and adults with CP spend approximately 76–99% of waking hours engaged in sedentary activities, with less than 18% spent on light physical activity (37). This is especially pronounced in individuals with moderate to severe impairments, for whom even light-intensity activities such as walking can be challenging, and who face additional barriers including pain, fatigue, and limited access to adapted physical activity options (33–35, 38).

Environmental and systemic barriers further restrict participation in exercise for individuals with CP. Families frequently encounter limited availability of adapted and community-based exercise programmes, insufficient transportation options, high equipment costs, and a lack of accessible information concerning local activities (38). Assistive devices such as adapted bicycles or running frames can facilitate participation but are often unavailable or expensive. The shortage of fitness and healthcare professionals trained to work with individuals with disabilities also represents a significant obstacle to safe and structured physical activity (38).

Physiological factors compound these barriers. Individuals with CP demonstrate a disproportionately high oxygen cost of movement (i.e., greater oxygen consumption per unit distance), meaning that even routine daily tasks such as self-care or transfers require substantially more energy than in typically developing peers (39–41). This elevated metabolic demand is central to the concept of the “physical activity paradox”: while physical activity is generally associated with positive health outcomes, obligatory activities performed for basic functioning may not confer the same benefits as voluntary, leisure-time exercise (42). Instead, the physiological cost of everyday tasks may induce persistent fatigue and deplete energy reserves, leaving little capacity for additional health-promoting physical activity (43–45). Over time, this may perpetuate a cycle of inactivity, reduced physical reserve, and progressive functional decline.

## CARDIORESPIRATORY ALTERATIONS INFLUENCING ENDURANCE CAPACITY

Endurance capacity relies on the integrated function of the cardiovascular and pulmonary systems, which, together with the oxygen-carrying capacity of the blood, ensure efficient oxygen delivery to working muscles (Fig. 1). The following sections explore cardiorespiratory and haematological factors that potentially contribute to low endurance capacity in individuals with CP.

**Fig. 1:**
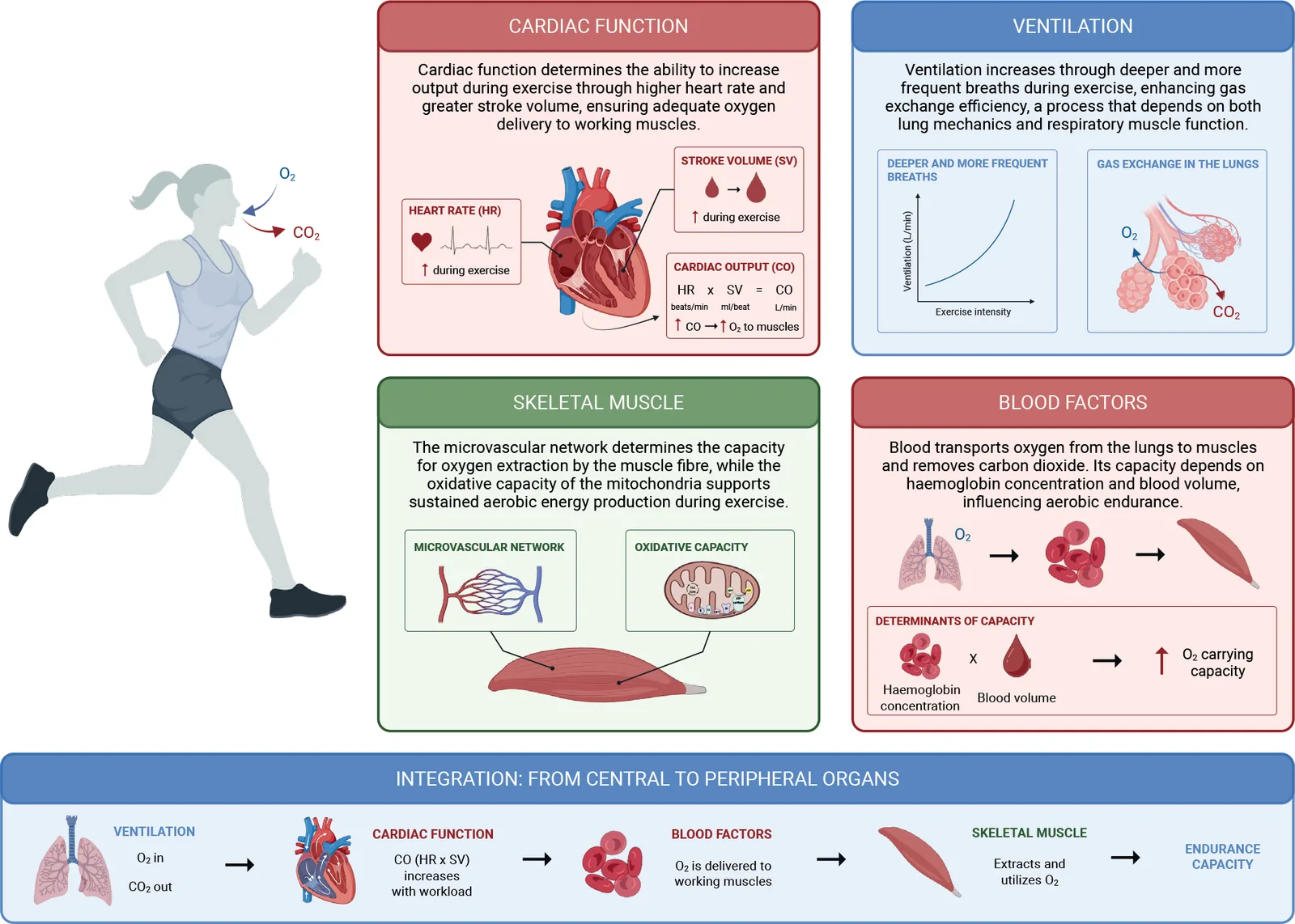
Physiological determinants of endurance capacity. Endurance capacity emerges from the integrated function of 4 interconnected physiological systems. Cardiac output – the product of heart rate and stroke volume, both of which increase progressively during exercise – governs the volume of oxygenated blood delivered to working muscles per minute. Simultaneously, ventilation increases through deeper and more frequent breaths, enhancing pulmonary gas exchange and facilitating uptake of oxygen (O_2_) while eliminating carbon dioxide (CO_2_). The oxygen-carrying capacity of the blood, determined by haemoglobin concentration and blood volume, dictates how much O_2_ can be transported from the lungs to peripheral tissues. At the muscular level, the microvascular network enables local oxygen delivery, while mitochondrial oxidative capacity sustains aerobic energy production. The bottom panel illustrates the integrated physiological cascade from pulmonary gas exchange through cardiac pumping and blood-borne transport to muscular extraction and utilization, collectively determining endurance capacity. CO_2_: carbon dioxide; CO: cardiac output; HR: heart rate; O_2_: oxygen; SV: stroke volume. Created in BioRender. Von Walden F. (2026) https://BioRender.com/6ims0cz.

### Cardiac physiology and function

Cardiac output, defined as the product of heart rate and stroke volume, is determined largely by cardiac structure and contractile function. These characteristics directly influence the efficiency of blood delivery to peripheral tissues during rest and exercise (46). However, cardiac structure and function have rarely been investigated in individuals with CP. Pastore et al. (47) performed echocardiographic and electrocardiographic assessments in young children with CP and found largely preserved cardiac morphology and resting function compared with typically developing peers, though some differences in cardiac conduction measures were noted – all within normal age-specific ranges. Given that these findings stem from a single study conducted at rest, conclusions regarding cardiac structure and function in individuals with CP should be drawn with caution.

### Autonomic regulation and the cardiovascular response to exercise

In addition to structural and resting cardiac considerations, the cardiovascular response to exercise relies on a finely regulated balance between the parasympathetic and sympathetic branches of the autonomic nervous system. Disruption of this balance can compromise exercise capacity through mechanisms such as inadequate regulation of blood pressure or heart rate (48).

Elevated resting heart rate, reported across several independent research groups in individuals with CP (49–55), suggests sustained sympathetic predominance at rest. This is further supported by reduced heart rate variability (HRV), a commonly used marker of autonomic function (56), reported in multiple studies (49, 51, 55). Some studies have reported lower HRV indices in individuals with more severe motor impairments (51, 55), though this association has not been consistently replicated (49). Taken together, these findings indicate increased sympathetic activity at rest in individuals with CP, with evidence suggesting greater autonomic dysregulation at higher levels of motor impairment.

This autonomic imbalance has direct consequences for the cardiovascular response during exercise. Elevated resting heart rate reduces the available heart rate reserve during exercise, constraining the chronotropic response to increasing workload. In independently ambulatory individuals with CP (GMFCS I–II), maximum heart rate during exercise has been reported to be within normal limits or only slightly reduced (27, 57–59). Among individuals with greater motor impairments (GMFCS III–IV), heart rate responses during submaximal and maximal exercise appear more attenuated (60, 61), though this may partly reflect reduced active muscle mass recruitment and lower metabolic demand, rather than autonomic dysregulation alone. Both HRV modulation during exercise and heart rate recovery following exercise, responses that are usually well preserved in typically developing individuals, appear impaired in individuals with CP (49, 54).

The integrative consequence of these cardiac and autonomic alterations may be reflected in oxygen pulse – calculated as VO_2peak_ divided by peak heart rate – which serves as a surrogate for the product of stroke volume and peripheral oxygen extraction efficiency (62, 63). Studies from several independent research groups have reported lower oxygen pulse values during exercise in both children and adults with CP (27, 29, 61), suggesting a meaningful limitation to endurance capacity under conditions of increased physiological demand. Whether this reduction is primarily driven by impaired stroke volume, reduced peripheral oxygen extraction, or both, remains to be determined.

While reduced physical activity and deconditioning are known to adversely affect cardiac remodelling (64), the autonomic dysregulation observed in individuals with CP may not be fully explained by inactivity alone. Elevated sympathetic activity and impaired HRV have been reported even in young children with CP, suggesting that neurological factors may contribute to these alterations early in life. The relative contributions of primary neurological mechanisms and secondary deconditioning remain to be clarified.

### Ventilatory limitations

Spirometry assessments consistently reveal reduced respiratory function in individuals with CP, with functional lung volumes typically measuring between 50% and 90% of those observed in typically developing individuals (61, 65, 66). More specifically, 1 study in children with spastic CP (GMFCS I–III) reported deficits of approximately 10–50% across key spirometric measures, including tidal volume, forced vital capacity (FVC), forced expiratory volume in 1 s (FEV_1_), and peak expiratory flow (66). The observation that FEV_1_/FVC ratios generally remain within normal ranges supports the presence of a primarily restrictive pattern, rather than obstructive pulmonary impairment (61, 65, 66).

During physical exertion, individuals with CP exhibit an altered breathing response. Work from several independent laboratories has shown disproportionately elevated respiratory frequencies during both submaximal and maximal exercise (29, 57, 61). For example, Balemans et al. (29) reported increased respiratory frequency during high-intensity exercise in children with CP (GMFCS I–III) using a cycle ergometer. A similar pattern was observed by Corell et al. (61) in adolescents and adults with CP (GMFCS II–IV) during treadmill-based testing with a running frame. These results suggest a reliance on increased breathing frequency to compensate for reduced functional lung volumes and respiratory efficiency during physical exertion. Interestingly, similar breathing patterns have been observed in Paralympic athletes with CP, suggesting that these limitations are not solely the result of inactivity or deconditioning, but may instead reflect an inherent feature of CP (57).

Ventilatory limitations in individuals with CP are likely multifactorial and arise from underlying neuromuscular, biomechanical, and anatomical impairments associated with the condition. At the neuromuscular level, respiratory muscle weakness and variations in muscle tone may limit the strength and coordination needed for effective ventilation (67). Supporting this, ultrasound-derived measurements have demonstrated reduced expiratory diaphragmatic excursion and shorter expiratory time in children with CP, suggesting impaired abdominal muscle coordination and trunk stabilization during expiration (68). These alterations appear more pronounced in nonambulatory individuals classified at GMFCS IV–V. Poor posture and reduced trunk control can further impair breathing mechanics by causing misalignment of the diaphragm and reducing its mechanical efficiency (67).

Biomechanical and anatomical factors may compound these neuromuscular impairments. Restrictions in chest wall mobility and thoracic expansion may further limit lung volumes during inspiration (67, 69, 70), often exacerbated by thoracic deformities such as scoliosis and kyphosis, which are highly prevalent in children and young adults with CP (71). Collectively, these impairments may lead some individuals with CP to rely on accessory respiratory muscles, increasing the mechanical work of breathing. Over time, this elevated respiratory workload not only contributes to respiratory fatigue, but may also divert oxygen delivery away from active locomotor muscles – directly limiting endurance capacity and contributing to premature termination of physical activity (72).

Given the multifactorial nature of these ventilatory limitations, several targeted interventions have been explored. Respiratory muscle training and breathing exercise interventions have been reported to improve respiratory muscle strength and lung volumes in individuals with CP (73, 74). Manual techniques targeting diaphragm mobility and chest wall expansion have also been investigated, though evidence remains limited and the mechanisms underlying potential effects are not fully understood (75). Although more research is needed to determine the optimal way to improve respiratory function, these interventions may not only improve physical performance but also mitigate the risk of developing respiratory comorbidities in individuals with CP (76).

### Oxygen transport and delivery

Haemoglobin is essential for oxygen transport in the blood, as it binds to oxygen in the lungs and delivers it to peripheral tissues. Despite its key role, haemoglobin levels in individuals with CP have not been systematically investigated. In a large cohort of children with CP, mean haemoglobin levels were around 115 g/L (77), which falls at the lower end of, or marginally below, age-specific ranges. The absence of age-matched typically developing peers in this study, however, precludes direct comparison. In addition, reported haemoglobin levels and anaemia prevalence in individuals with CP vary across studies, with evidence from both children and adults showing inconsistent findings (61, 77, 78).

Malnutrition, including micronutrient deficiencies such as low iron, is common in individuals with CP (79–81). Iron deficiency may limit haemoglobin synthesis, potentially reducing oxygen-carrying capacity, which in turn could influence endurance performance. Orhan et al. (81) reported lower circulating iron levels alongside higher total iron-binding capacity in individuals with CP compared with typically developing peers, findings consistent with iron deficiency. Whether this translates to clinically relevant reductions in haemoglobin and impaired oxygen transport remains uncertain. Similarly, plasma volume, which together with haemoglobin concentration determines total oxygen-carrying capacity, has not been investigated in individuals with CP, representing a notable gap in the current literature. Nevertheless, studies measuring oxygen saturation have generally reported normal values, despite reduced functional lung volumes and restricted chest wall expansion (82). Supporting this, a recent study using near-infrared spectroscopy in adults with CP found that muscle oxygen saturation was higher than in typically developing controls across various exercise intensities, indicating adequate oxygen delivery to working muscles, while also suggesting that peripheral oxygen extraction, rather than delivery, may represent the primary limiting factor (83).

Taken together, current findings suggest that although mild deviations in iron or haemoglobin status may be present, impaired oxygen transport or delivery does not appear to play a major role in limiting endurance capacity in individuals with CP.

## PERIPHERAL LIMITATIONS OF ENDURANCE CAPACITY IN INDIVIDUALS WITH CEREBRAL PALSY

Endurance capacity depends not only on oxygen delivery from the cardiopulmonary system, but also on the ability of skeletal muscle to extract and utilize oxygen. In individuals with CP, combined cellular and structural alterations impair the muscles’ capacity for oxygen utilization and force production. Consequently, aerobic performance may be compromised even when oxygen delivery is sufficient (84, 85). These changes are believed to stem from both the underlying neurological condition and deconditioning secondary to reduced activity levels.

### Microscopic level

Capillary density is a central factor for efficient oxygen diffusion from the microvasculature to the individual muscle cells (i.e., myofibres) (86). The capillary network in skeletal muscle is highly adaptable and can expand through angiogenesis in response to endurance training (86, 87), but may also undergo capillary rarefaction during periods of physical inactivity (88, 89). Individuals with CP have been shown to have fewer capillaries surrounding each muscle fibre compared with typically developing individuals (90, 91). This reduced capillary density limits the total surface area available for oxygen diffusion and may constrain oxygen availability during periods of increased metabolic demand. Despite the lower number of capillaries, the ability of the remaining vessels to dilate and increase blood flow during exercise appears preserved (59). This vasodilatory capacity is mediated in part through vasoactive molecules such as nitric oxide (92), suggesting that the primary limitation is related to capillary number rather than acute regulation of blood flow.

At the intracellular level, mitochondrial function represents a further critical determinant of aerobic capacity. Mitochondria facilitate oxidative phosphorylation through the electron transport chain (ETC), enabling efficient adenosine triphosphate (ATP), i.e., energy production. Here, electrons derived from nutrient metabolism are passed along the chain of proteins in the ETC through a series of redox reactions, ultimately driving the synthesis of ATP (93, 94). Evidence indicates that mitochondrial function is impaired in skeletal muscle of individuals with CP. This is reflected by markedly reduced expression of gene transcripts and proteins that constitute the ETC (95, 96), along with reduced enzymatic activity of key complexes (I, II, and III) by as much as 45–80% compared with typically developing muscles (97). Consequently, the capacity for oxidative ATP production is likely to be diminished, limiting the efficiency of aerobic metabolism. Supporting this, non-invasive measurements using near-infrared spectroscopy show that skeletal muscle in individuals with CP extracts significantly less oxygen from the circulation, both at rest and during contraction, consistent with the higher muscle oxygen saturation described in the preceding section (83).

When aerobic energy production cannot meet the metabolic demand, the muscle increasingly relies on anaerobic pathways, which generate less ATP per unit of substrate and accelerate the accumulation of metabolic by-products. This metabolic shift may not only accelerate fatigue but also increase the perceived effort during physical activity (98, 99), potentially contributing to the lower endurance capacity commonly observed in children and adults with CP (25–29).

Although these findings highlight limitations in both oxygen diffusion and utilization, the extent to which skeletal muscle in individuals with CP can adapt to endurance training at the cellular level remains largely unknown. While training interventions, including Frame Running – a sport using a three-wheeled running frame equipped with a saddle and handlebars that enables self-propelled locomotion with postural support – have demonstrated improvements in cardiorespiratory fitness and muscle hypertrophy (100), adaptations specifically at the level of capillarization and mitochondrial biogenesis have yet to be examined in this population. Given the magnitude of the deficits described above, such cellular adaptations could have meaningful implications for endurance capacity and long-term metabolic health.

### Macroscopic level

Skeletal muscle size and architecture not only determine force production capacity, but also contribute to endurance by influencing the relative metabolic demand of physical activity. A given task requires less relative effort, and can therefore be sustained for longer, when performed by a larger muscle operating at a lower percentage of its maximal capacity. Key indicators such as muscle volume, cross-sectional area (CSA), and muscle thickness are therefore relevant for both strength and endurance performance (101, 102).

In individuals with CP, skeletal muscle characteristics at the macro level are often compromised due to neurological and developmental factors. Skeletal muscle growth is slower in children with CP, and adults with CP generally exhibit lower skeletal muscle mass, likely driven by a combination of motor impairments and sedentary lifestyle associated with CP (103). These alterations are reflected in reduced muscle belly length, volume, CSA, and muscle thickness compared with typically developing individuals (104–106). Muscle thickness also correlates positively with functional level in children with CP (107). Furthermore, increased tendon length together with reduced tendon CSA restrict force production and maximum muscle power output (105, 108, 109). These structural changes contribute to significant deficits in muscle strength, functional performance, and gait (110–114). Parameters of muscle architecture, such as muscle thickness and fascicle length, also correlate significantly with muscle strength and force production in individuals with CP (115). However, the relationship between muscle morphology and function may be further complicated by neurological impairments such as spasticity, poor motor control, and reduced central drive, resulting in strength levels that are disproportionately low relative to muscle size (116).

Although greater muscle size is associated with enhanced strength and force production, endurance capacity also depends on metabolic muscle characteristics such as mitochondrial density and capillarization (117). Thus, while adequate muscle mass contributes to the ability to sustain physical activity, structural adaptations alone are unlikely to fully explain or improve endurance capacity in individuals with CP.

## TESTING MODALITIES FOR INDIVIDUALS WITH CEREBRAL PALSY

Assessing cardiorespiratory fitness in individuals with CP presents unique challenges due to the heterogeneity of the condition and the variability in motor function and mobility limitations across different GMFCS levels. Impaired motor control, postural instability, coordination difficulties, and/or cognitive limitations can often complicate the use of traditional exercise testing protocols, particularly at higher GMFCS levels where these limitations are more pronounced. Therefore, careful selection of testing modalities is essential to ensure that assessments are safe, valid, and feasible.

Assessment methods can be broadly categorized according to testing context (laboratory-based vs field-based) and effort level (maximal vs submaximal) and should be selected based on functional ability of the individual. In the following section, we introduce endurance capacity tests used in research for individuals with CP. Where appropriate, adaptations for different GMFCS levels are also highlighted.

### Laboratory-based maximal effort tests

Laboratory-based assessment of cardiorespiratory fitness involves progressive incremental exercise protocols usually performed on a treadmill or cycle ergometer, with continuous breath-by-breath gas exchange analysis used to calculate VO_2_ and respiratory exchange ratio. True maximal oxygen uptake (VO_2max_) requires a demonstrable plateau in oxygen uptake despite increasing workload, a criterion that can be challenging to achieve. As an alternative, VO_2peak_, defined as the highest recorded VO_2_ during an incremental test, is commonly used.

Treadmill protocols have primarily been applied to ambulatory individuals with CP (GMFCS I–II) (25, 27, 118–122), whereas cycle ergometry has been used across GMFCS I–III (26, 123–131). A smaller number of studies have employed arm crank ergometry to enable testing of wheelchair users (125, 131–133).

More recently, Hjalmarsson et al. (134) introduced an alternative protocol to improve accessibility for individuals with limited mobility: the Frame Running Incremental Treadmill Test (FRITT), which utilizes a running frame on a wide treadmill. Although evidence remains limited, this adaptation may allow individuals with CP to perform maximal-effort testing with greater stability and support. FRITT thus represents a promising method for extending maximal testing to individuals at higher GMFCS levels who may not otherwise tolerate conventional treadmill or cycling protocols.

### Laboratory-based submaximal effort tests

Even with adaptations for maximal laboratory testing, achieving maximal physiological exertion may remain challenging for some individuals with CP. As a result, the highest VO_2_ achieved during testing may not reflect the individual’s true VO_2max_. In these situations, submaximal testing protocols may provide a practical alternative.

Submaximal tests typically use treadmill or cycle ergometry to estimate VO_2peak_ without requiring exhaustion. Although treadmill-based submaximal protocols have been employed in individuals with CP (GMFCS II–V), including work from our research group, these have primarily been used to assess physiological and ventilatory responses rather than to predict VO_2peak_ (61). Instead, submaximal cycle ergometry has demonstrated acceptable validity for predicting VO_2peak_ in individuals with CP (127). Although less precise than direct maximal testing, submaximal protocols are generally safe and accessible, and provide meaningful information on endurance capacity for those unable to reliably perform maximal tests.

### Field-based maximal effort tests

Field-based maximal tests provide a feasible alternative for estimating VO_2peak_, requiring minimal equipment and allowing administration in diverse settings. Rather than directly measuring VO_2_, these tests assess maximal performance outcomes (e.g., distance or stages completed) and use these as indirect indicators of cardiorespiratory fitness.

The Six-Minute Frame Running Test (6-MFRT) (135) enables individuals using a running frame (typically GMFCS II–IV) to perform high-intensity effort over 6 min. The 6-MFRT demonstrates high test–retest reliability (136), and the distance covered correlates strongly with VO_2peak_, supporting its effectiveness as an indicator of cardiorespiratory fitness (134). Although performed at a self-selected pace, most participants achieve sufficient exertion for the 6-MFRT to be considered a valid maximal field assessment (134, 137).

Shuttle run tests similarly provide valid and reliable measures of cardiorespiratory fitness for ambulatory individuals (GMFCS I–II) (119). Adapted shuttle run protocols exist for individuals at GMFCS III (138); however, further validation is needed to establish their reliability and validity in this group.

### Field-based submaximal effort tests

Field-based submaximal assessments offer the advantage of feasibility and relevance to real-world activities. The Six-Minute Walk Test (6-MWT) evaluates endurance without requiring maximal effort, making it accessible to individuals across a broader range of motor abilities (126, 139, 140). While reliability is generally acceptable, the validity of 6-MWT as a measure of cardiorespiratory fitness is limited, particularly in higher-functioning individuals (141). Nevertheless, the test provides clinically meaningful information on functional mobility and is widely used in both research and clinical settings.

Collectively, these assessment approaches provide a structured basis for evaluating endurance capacity in individuals with CP. In the next section, we briefly discuss how such assessments can inform practical training strategies and support physical activity participation.

## IMPLICATIONS FOR TRAINING AND PRACTICE

Current WHO guidelines recommend that children and adolescents accumulate at least 60 min per day of moderate- to vigorous-intensity aerobic activity, including vigorous-intensity and muscle- and bone-strengthening activities on at least 3 days per week. For adults, guidelines recommend 150–300 min of moderate-intensity aerobic activity per week – or 75–150 min of vigorous-intensity, combined with muscle-strengthening exercise on 2 or more days per week, alongside minimizing sedentary behaviour to reduce cardiometabolic risk (142). However, achieving these recommendations may be particularly challenging for individuals with CP due to substantial variation in functional abilities and exercise tolerance (9, 33, 34, 143).

The FITT principle (Frequency, Intensity, Time, and Type) provides a useful guide for structuring training in individuals with CP (144). Given the often elevated energy cost of movement and the presence of fatigue in this population, it may be necessary to introduce physical activity gradually, adapting the activity and tailoring intensity to individual tolerance. Even modest increases in activity can confer meaningful physical and mental health benefits (142, 145), and replacing sedentary behaviour with light-intensity activity, even in short bouts, is a realistic and effective first step. For continuous improvements, appropriate progression of intensity, volume, and duration over time should be applied (144, 146).

The peripheral and ventilatory findings described in this review further highlight that endurance training alone is unlikely to be sufficient. Resistance training is needed to tackle structural muscle deficits, and respiratory muscle training may complement aerobic exercise by improving ventilatory capacity. For individuals with autonomic dysregulation, heart rate-based intensity may be less reliable; perceived exertion scales, breathing patterns, or workload measures may therefore provide more accurate guidance. Given the impaired heart rate recovery and altered HRV responses, adequate recovery time between sessions should also be considered to avoid cumulative fatigue.

Encouragingly, individuals with CP improve endurance capacity with training (146, 147). Adaptive cycling has demonstrated benefits in cardiorespiratory fitness, autonomy, and psychosocial well-being, though evidence on long-term outcomes and equipment types remains limited (148). Frame Running is feasible and effective across a wide functional range, with studies reporting improvements in cardiorespiratory fitness, muscular strength, and participation (100, 149–152). These modalities reduce balance demands and likely reduce the relative energy cost of movement, enabling participants to achieve higher intensities and longer durations than during unsupported gait. As such, they represent important tools for expanding aerobic training opportunities while supporting both health-related and psychosocial outcomes.

Beyond physiological considerations, prioritizing activities that are meaningful, enjoyable, and socially engaging is important. The “F-words” in childhood disability (function, family, fitness, fun, friends, future) provide a practical framework to align goals with motivation and context (153). Integrating these principles may enhance adherence to physical activity recommendations and support long-term engagement in physical activity. Inclusive infrastructure, community programmes, and access to trained professionals can also remove barriers and promote sustained engagement (38, 154).

## CONCLUSION

Individuals with CP consistently exhibit reduced endurance capacity compared with typically developing peers, due to a combination of central and peripheral physiological factors (Fig. 2). Evidence on cardiac structure in individuals with CP remains limited, and further research is needed to clarify whether structural differences affect cardiovascular function during exercise. Altered autonomic regulation and lower oxygen pulse may further constrain exercise responses, particularly at higher GMFCS levels. Pulmonary limitations, including restrictive breathing patterns reflected in reduced functional lung volumes, and compromised chest wall mobility, further challenge ventilatory efficiency during activity. At the peripheral level, skeletal muscles in individuals with CP exhibit reduced capillary density, reduced mitochondrial function, decreased muscle volume, and architectural changes, collectively impairing oxygen utilization and force generation, and compounding limitations in endurance performance. Despite reported variations in haemoglobin or iron status in some individuals with CP, evidence suggests that impaired oxygen transport or delivery is not the primary driver of reduced endurance capacity – rather, peripheral muscle alterations and metabolic dysfunction appear central.

**Fig. 2:**
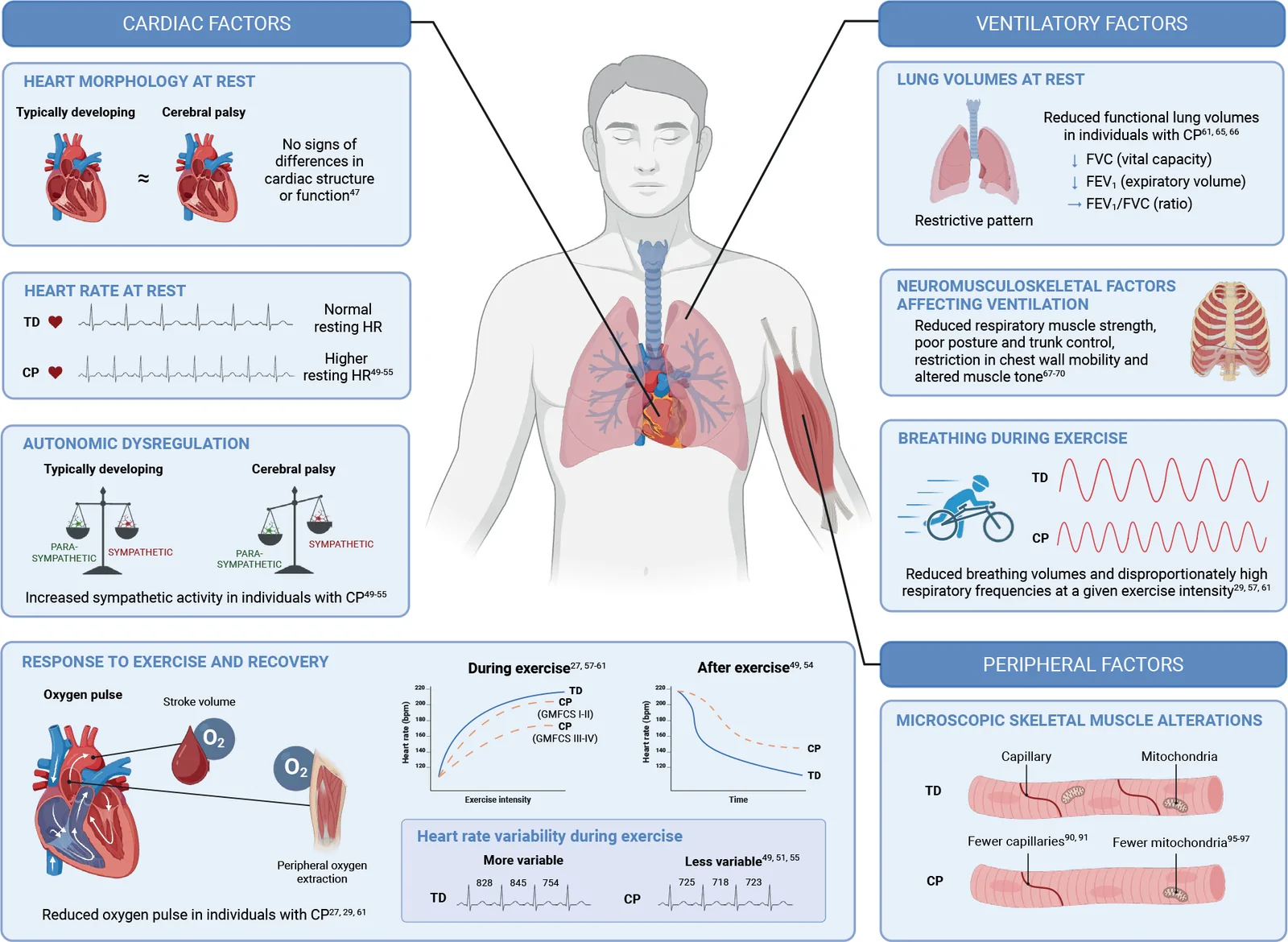
Physiological alterations in individuals with cerebral palsy (CP) compared with typically developing peers. Individuals with CP exhibit several resting and exercise-related physiological differences compared with typically developing peers. Resting heart rate (HR) is elevated in individuals with CP, reflecting autonomic dysregulation characterized by increased sympathetic and reduced parasympathetic activity. During exercise, heart rate variability is reduced, and post-exercise recovery is attenuated – collectively indicating diminished cardiac autonomic flexibility. Oxygen pulse, a surrogate for the product of stroke volume and peripheral oxygen extraction, is reduced in individuals with CP. Ventilatory function is also altered with a restrictive pattern at rest (i.e., reduced forced vital capacity and forced expiratory volume in 1 s) and are believed to be attributed to reduced respiratory muscle strength, reduced coordination, poor posture and trunk control, restricted chest wall mobility, and altered muscle tone. During exercise, breathing volumes are reduced and respiratory frequencies are disproportionately elevated relative to exercise intensity. At the peripheral level, skeletal muscle in CP is characterized by reduced capillary density and fewer mitochondria, impairing both microvascular oxygen delivery and oxidative metabolic capacity. bpm: beats per minute; FEV1: forced expiratory volume in 1 s; FVC: forced vital capacity; GMFCS: Gross Motor Function Classification System; TD: typically developing. Created in BioRender. Von Walden F. (2026) https://BioRender.com/6ims0cz

Combined with low physical activity, high sedentary time, and the elevated energy cost of movement, these physiological impairments collectively underscore the multifactorial nature of endurance limitations in individuals with CP.

Promisingly, several interventions have demonstrated benefits across functional levels. Aerobic training, resistance exercise training, and task-specific modalities such as running frames and adaptive cycling have shown improvements in physiological capacity and participation. These findings emphasize the need for individually tailored approaches that accommodate the heterogeneity of CP to enhance functional endurance and overall health.

Future work should clarify the relative contribution of central vs peripheral limitations, characterize the long-term adaptations to different training modalities at both central and peripheral levels, and identify strategies that sustain physical activity across the lifespan. A deeper mechanistic understanding will refine intervention design and help promote better participation, health, and quality of life for individuals with CP.
